# Editorial: Hypoxia and inflammation: A two-way street

**DOI:** 10.3389/fimmu.2023.1171116

**Published:** 2023-03-08

**Authors:** Philippe Saas, Guo-Chang Fan

**Affiliations:** ^1^ EFS, Recherche et Développement, Grenoble, France; ^2^ Université Grenoble-Alpes, INSERM U1209, CNRS UMR 5309, Institute for Advanced Biosciences, Grenoble, France; ^3^ Department of Pharmacology & Systems Physiology, University of Cincinnati College of Medicine, Cincinnati, OH, United States

**Keywords:** hypoxia, HIF, inflammation, macrophages, hematopoietic progenitor and stem cells, inflammatory diseases, efferocytosis

Oxygen homeostasis is crucial for survival, and mammals have developed fine regulatory mechanisms in response to oxygen variations. The role of oxygen availability in physiological and pathological processes catches more and more attention. Oxygen tension in mammalian body varies depending on the considered tissue. An oxygen gradient exists between the air we breathe (~21% O_2_), present in lung alveoli, and the oxygen tension found in other mammalian tissues. Accordingly, normoxia is tissue-dependent. Within a given tissue, oxygen distribution varies. Indeed, the partial oxygen pressure (PO_2_) of the bone marrow (BM) -the primary site of hematopoiesis- is different in the human sternum and iliac crest marrow. Reduced oxygen availability -a situation called physiological hypoxia- is detected in localized areas in the BM that are critical for hematopoietic stem and progenitor (HSPC) differentiation. For a same organ such as the spleen, PO_2_ values differ from one species to another, reflecting the heterogeneous perfusion of this secondary lymphoid organ. Besides the physiological variations of oxygen availability, pathological hypoxia is a common hallmark of several inflammatory diseases such as cancers and infectious diseases. *In vitro* experiments performed in standard cell culture incubators (5% CO_2_, 75% humidity) should be considered as hyperoxic conditions (~18.5% O_2_) for cultured cells. Therefore, the terms hyperoxia, normoxia, and hypoxia should be used contextually rather than absolutely, since oxygenation is variable *in vivo*. For better interpretation, oxygenation should always be defined quantitatively (1).

The main cellular oxygen sensors are hypoxia-inducible factors (HIFs) with HIF-1α being the most studied transcription factor. Activation of HIF-1α by hypoxia leads to its translocation to the nucleus (for details on HIF-1α activation, refer to Thomas et al.). After translocation, HIF-1α binds to hypoxia-response elements, which initiate the transcription of hypoxia-sensitive genes. These genes code for different proteins (e.g., vascular endothelial growth factor [VEGF], erythropoietin [EPO], or glucose transporter 1) decreasing cellular oxygen consumption and/or increase oxygen delivery (1). Hypoxia influences immune cell functions by regulating metabolic pathways, and can be a pathogenic factor in some inflammatory diseases. Conversely, inflammation can lead to local hypoxia. The aim of this Research Topic was to gather articles discussing/studying the relationship between hypoxia and inflammation. This topic collects five original research manuscripts and one review dealing with six different diseases associated with hypoxia and inflammation. Four diseases affect the lungs: sarcoidosis (Jeny et al.), chronic obstructive pulmonary disease (COPD), obstructive sleep apnea (Florentin et al.) as chronic diseases and coronavirus disease 2019 (COVID-19) (Diaz-Garcia et al.) as an acute disease. Atherosclerosis (Thomas et al.) and myocardial infarction (MI) (Qi et al.) target the cardiovascular system. Several cell types are exposed to hypoxia, including a rat myoblast cell line (Qi et al.), human circulating leukocytes (Diaz-Garcia et al.), mouse and human HSPC (Florentin et al.) and macrophages (Thomas et al.; Emam et al.; Jeny et al.).

The *in vitro* oxygen-glucose deprivation (OGD) model is utilized for the culture of H9c2, a myoblast cell line derived from embryonic rat heart, in a sugar-free medium under hypoxic conditions (1% O_2_, 12 hours). Qi et al. show that OGD induces NLRP3 inflammasome activation; whereas treatment of H9c2 cells with ginsenoside-Rh2 (a Chinese medicine compound) and exosomes collected from mesenchymal stem cells reduces this activation. This may represent a new therapeutic approach for the reduction of ischemia-induced cardiac inflammation.

Diaz-Garcia et al. report that circulating soluble CD39 increases in patients developing a severe form of COVID-19. This increase is associated with increased CD39 expression on circulating T and NK cells, but also with hypoxemia severity and clinical prognosis. *In vitro* experiments using peripheral blood-derived mononuclear cells (PMBCs) cultured under hypoxic conditions (9% O_2_, 16 hours) confirm this enhanced expression of CD39 on T and NK cells, while decreased expression of CD73 is observed. CD73 is responsible for the final degradation of adenosine triphosphate and diphosphate into the immunosuppressive adenosine (2). Accumulation of these two adenosine nucleotides resulting from altered CD73 expression stimulates purinergic receptors expressed by platelets and monocytes. This leads to platelet and monocyte activation inducing both thrombus formation and inflammatory cytokine production. These results are recently confirmed by others (3, 4). In severe COVID-19, hypoxia could be responsible for uncontrolled thrombo-inflammation.

In two mouse models -mice exposed to 10% O_2_ for three weeks and the cigarette smoke-induced COPD model-, Florentin et al. determine the effects of chronic hypoxia on HSPC proliferation. Hypoxia induces HSPC proliferation *via* the upregulation of VEGF and its receptor, VEGF receptor 1 (VEGFR1). *HIF1A* silencing in both human and mouse HSPC reduces hypoxia-induced proliferation and hypoxia-induced *VEGFR1* mRNA expression. *VEGFR1* is thus another HIF-1α target gene. Furthermore, inhibiting the VEGF/VEGFR1 axis could limit hypoxia-induced inflammation.

Macrophages, a heterogeneous cell population with a high plasticity, may arise from HSPCs during embryogenesis to become tissue-resident macrophages. Alternatively, during inflammation, macrophages are differentiated from monocytes (MDMs) (5). Macrophages exert a vast range of functions characterized by an array of phenotypes with two extreme polarized phenotypes, M1 and M2 (schematically pro-inflammatory and anti-inflammatory/resolving macrophages) (6). HIF1α-dependent glycolysis favors the M1 phenotype, while M2 macrophages seems to be HIF-independent (7). Thomas et al. discuss the bidirectional interaction between hypoxia/HIF-1α and cholesterol metabolism in atherosclerosis. In atherosclerotic plaques, cholesterol engulfed by macrophages trigger reactive oxygen species (ROS) synthesis, responsible for HIF-1α activation. The liver X receptor pathway stimulated by cholesterol-derived oxysterols may interact directly with HIF-1α. Conversely, hypoxia and HIF-1α favor the accumulation of cholesterol in macrophage by increasing its uptake and limiting its efflux. Hypoxia induces the accumulation of free cholesterol –a pro-inflammatory trigger– in advanced atherosclerotic plaques.

Jeny et al. investigate the role of hypoxia in M-CSF-induced human MDMs. Monocytes obtained from patients with pulmonary sarcoidosis and healthy controls are differentiated, and exposed to hypoxia (1.5% O_2_, 24 hours). Exposure of MDMs from patients with active sarcoidosis (AS) to hypoxia activates HIF-1α and pro-inflammatory cytokine synthesis without activating the NF-κB pathway. Hypoxia confers also to MDMs of AS patients, a pro-fibrotic profile with the increase of pro-fibrotic factors (e.g., VEGF-A, and plasminogen activator inhibitor-1 [PAI-1]). This mixed pro-inflammatory/pro-fibrotic profile induced by hypoxia contrasts with the mild pro-fibrotic profile observed in MDMs from healthy donors. Expression of HIF-1α and PAI-1 in the nucleus of macrophage-derived epithelioid cells in pulmonary biopsies of AS patients supports the clinical relevance of these findings. Comparing atmospheric (~21% O_2_) to hypoxic conditions (1.5% O_2_) is appropriate here; physiologically, lung macrophages are exposed to atmospheric conditions. Contrarily, sarcoidosis granulomas are hypoxic (8). In contrast to M-CSF that generates less differentiated MDMs, GM-CSF promotes a pro-inflammatory phenotype in MDMs (9). Emam et al. determined the impact of host genetics (appreciated by single nucleic polymorphisms [SNP]) on the ability of GM-CSF-induced bovine MDMs to produce nitric oxide (NO) in response to *Escherichia coli*. Among the 43,066 SNPs studied, 60 SNPs of the bovine genome were statistically associated with NO production. Four genes belong to the Gene Ontology term “response to hypoxia”. The authors speculate that modulation of these genes is indirectly related to hypoxia, but linked to respiratory/oxidative burst (*i.e*., the fast release of the ROS). Indeed, this burst generates hypoxia at the macrophage level and activates HIF-1α (10). This last work is interesting for this editorial, since respiratory burst-induced hypoxia activates macrophage EPO signaling to promote inflammation resolution. This burst induces a local hypoxia that activates HIF-1α. HIF-1α activation leads to EPO secretion that stimulates EPO receptor in an autocrine manner. EPO pathway increases apoptotic neutrophil elimination (the efferocytosis process) promoting the resolution phase of inflammation (10). Efferocytosis is critical, since neutrophils play a major role in depleting local oxygen in inflamed tissue (2). Chronic hypoxia increases efferocytic capacities of both murine and human macrophages (11, 12). Thus, hypoxia could also promote inflammation resolution (2).

In conclusion, this Research Topic provides additional information on the relationship between hypoxia and inflammation.

## Author contributions

All authors listed have made a substantial, direct and intellectual contribution to the work and approved it for publication.
